# Biodiversity of strains belonging to the freshwater genus *Aquirufa* in a riparian forest restoration area in Salzburg, Austria, with a focus on the description of *Aquirufa salirivi* sp. nov. and *Aquirufa novilacunae* sp. nov

**DOI:** 10.1007/s10123-025-00642-x

**Published:** 2025-02-18

**Authors:** Alexandra Pitt, Stefan Lienbacher, Johanna Schmidt, Meina Neumann-Schaal, Jacqueline Wolf, Hannah Wenng, Aharon Oren, Zoe Huber, Martin W. Hahn

**Affiliations:** 1 Research Department for Limnology, Universität Innsbruck, 5310 Mondsee, Austria; 2 Metabolomics and Services, Leibniz Institute DSMZ-German Collection of Microorganisms and Cell Cultures GmbH, Braunschweig, Germany; 3 Haus der Natur, Museum für Natur und Technik, 5020 Salzburg, Austria; 4 The Institute of Life Sciences, The Hebrew University of Jerusalem, The Edmond J. Safra Campus, 9190401 Jerusalem, Israel

**Keywords:** *Aquirufa*, *Spirosomataceae*, Freshwater bacteria, Metagenome, Genome size, Riparian forest, Citizen science

## Abstract

During a citizen science project, four freshwater habitats in a riparian forest restoration area in Salzburg, Austria, were sampled. The primary aim was to obtain bacterial strains of the genus *Aquirufa*, a group of typical and widespread freshwater bacteria. Numerous pure cultures of *Aquirufa* strains could be obtained, three of them originating from the river Salzach, a newly created pond and the lake Ausee represented new species. Strain 1-SAACH-A3^T^ was characterized by a genome size of 3.2 Mbp and a G + C value of 38.4 mol% and encoded genes predicted for nitrate uptake and nitrous oxide utilization. Strains BAHN-186B^T^ and 2-AUSEE-184A6 were characterized by a genome size of 2.4 Mbp and a G + C value of 42.4 and 42.2 mol%, respectively, and encoded genes predicted for the light-harvesting rhodopsin system. Calculated whole-genome average nucleotide identity values with *Aquirufa* type strains resulted in a maximum value of 93.65% for comparison of strain 1-SAACH^T^ with the type strain of *Aquirufa ecclesiirivi*, which is slightly under the proposed threshold of species demarcation. The calculated gANI value comparing strains BAHN-186B^T^ and 2-AUSEE-184A6 revealed 95.76%, thus a value slightly above the threshold. Further analyses revealed that the three new strains represent two new species, proposed here as *Aquirufa salirivi* sp. nov. with type strain 1-SAACH-A3^T^ (= DSM 117800^ T^ = JCM 37097^ T^) and *Aquirufa novilacunae* sp. nov. with type strain BAHN-186B^T^ (= DSM 118143^ T^ = JCM 37099^ T^). Analyses of 123 publicly available metagenomes and a metagenome of the lake Ausee resulted in no detection of *A. salirivi* sp. nov. In contrast, *A. novilacunae* sp. nov. could be detected in 15 water samples of rivers, mainly from Asia, but also from North America and Australia. The analyses suggested that the species occurs in most of these samples in low relative abundance, detections derived from metagenomes of water samples from the river Yangtze in the subtropical zone could be interpreted as occurrence in higher abundances.

**Supplementary Information:**

The online version contains supplementary material available at 10.1007/s10123-025-00642-x.

## Introduction

The genus *Aquirufa* was established in 2019 (Pitt et al. 2019) and encompasses currently eight species with validly published names (Pitt et al. 2019, 2020, 2022, 2024; Sheu et al. 2020). The genus represents a group of typical and widespread freshwater bacteria. It belongs to the order *Cytophagales* (phylum *Bacteriodota*) and was originally assigned to the family *Cytophagaceae* (Pitt et al. 2019). Whole-genome-based phylogenetic analyses showed polyphyly of the family *Cytophagaceae* (García-López et al. 2019), and some authors proposed a reorganization of the taxon. Currently, the situation remains unsatisfactory, because the LPSN Database (Parte 2013) assigns the genus *Aquirufa* to the family *Spirosomataceae* while the NCBI Taxonomy database (Schoch et al. 2020) follows Lu et al. (2024) who created the new family *Flectobacillaceae* including the genus *Aquirufa* along with four further genera. Genome-based phylogenetic trees showed that the genus *Aquirufa* forms two distinct branches, the *A. antheringensis* branch and the *A. nivalisilvae* branch (Pitt et al. 2024). The species of the *A. antheringensis* branch have slightly smaller genome sizes and higher genomic G + C values than the species of the *A. nivalisilvae* branch. Typical for all *Aquirufa* strains are genome sizes around 3 Mbp and genomic G + C values around 40 mol%, red pigmentation, and rod-formed cells; they grow aerobically and chemoorganotrophically.

Within a citizen science project, freshwater habitats in a riparian forest restoration area in Salzburg, Austria, were sampled over 1 year with the primary aim of obtaining bacterial isolates belonging to the genus *Aquirufa*. This area called “Weitwörther Au” is a part of the riparian area of the river Salzach (Salzachauen) which is included in the Natura 2000 network of protected areas. It is located north of the city of Salzburg and covers an area of 3 km^2^. From 2015 to 2021, the Weitwörther Au was the focus of a renaturation project, aiming to reestablish natural conditions (Land-Salzburg 2021). Historically, this riparian area was used for intensive agriculture, forestry (specifically spruce monoculture), and gravel and sand extraction. Approximately 20 years ago, sand mining led to the creation of an artificial lake, the Ausee, which had been used for fishing. The main goal of the renaturation was to recreate various typical natural habitats through the conversion of forests, the establishment of ponds fed by groundwater and precipitation and the revitalizing of the water bodies like tributaries of the Salzach and the quarry lake.

The sampling of four selected water bodies led to several pure cultures assigned to the genus *Aquirufa*. Besides strains belonging to known *Aquirufa* species, some isolates represented new species. Strain 1-SAACH-A3^T^ originated from a water sample taken from the river Salzach in the summer of 2023 and 2-BAHN-186B^T^ and 2-AUSEE-184A6 from water samples taken in autumn 2023 in the restoration area beside the river from a small pond and the lake, respectively. Genome sequencing and calculated whole-genome average nucleotide identity values (gANI) revealed that strain 1-SAACH-A3^T^ represents a new species within the *A. nivalisilvae* branch, for which we propose the name *Aquirufa salirivi* sp. nov. Strains 2-BAHN-186B^T^ and 2-AUSEE-184A6 belonging to the *A. antheringensis* branch of the genus were of special interest. Calculated overall genome-related indexes were very close to demarcation values separating two species but revealed that the two strains represent only a single new species, for which we propose the name *Aquirufa novilacunae* sp. nov. with 2-BAHN-186B^T^ as the type strain.

## Materials and methods

### Sampling and isolation of strains

**Fig. 1:**
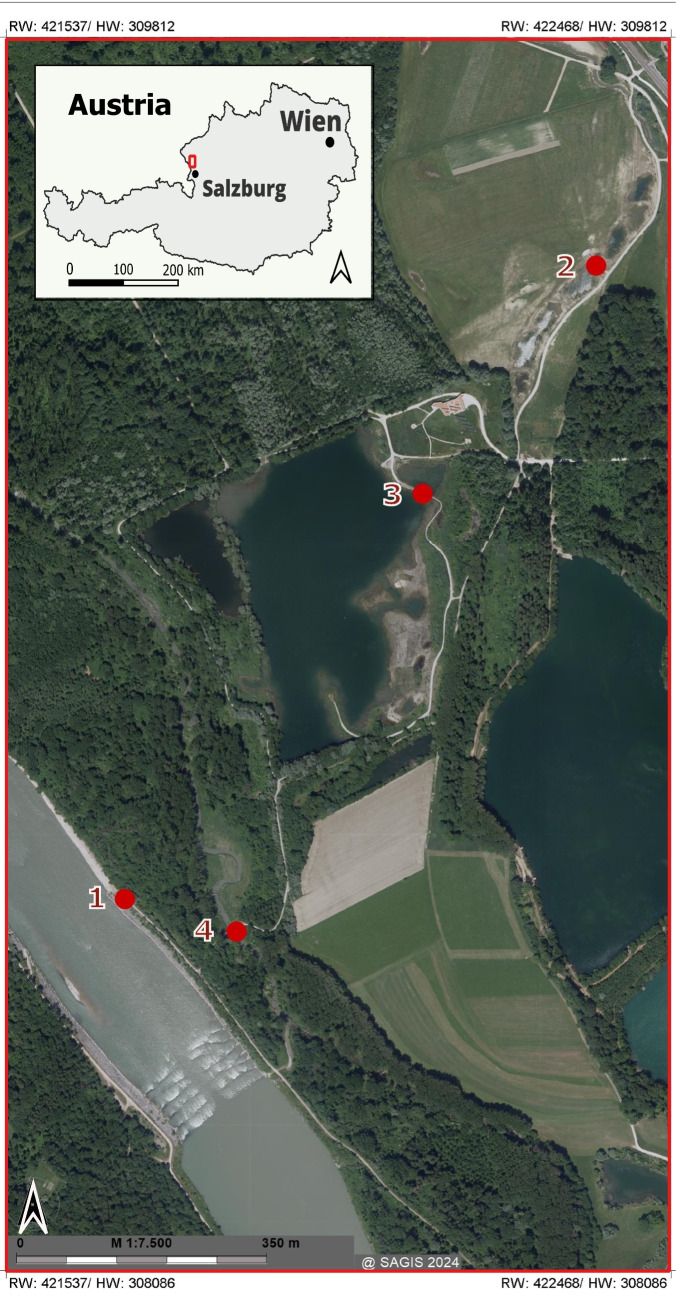
Map of the sampled habitats in the riparian forest restoration area Weitwörther Au. 1, river Salzach; 2, newly created pond; 3, lake Ausee; 4, creek Reitbach

Within the riparian forest restoration and wetland area “Weitwörther Au,” we chose four sampling sites, the river Salzach, the creek Reitbach, the lake Ausee, and a newly created small pond. Figure 1 shows the study area and was generated using SAGIS a geographical information system provided by the province/federal state of Salzburg (https://www.salzburg.gv.at/sagis). Water samples were taken from approximately 0.1-m depth, and measurements for pH, electrical conductivity, water temperature, oxygen concentration, and saturation were performed on location with a WTW multiparameter probe. The water samples were filtered as soon as possible through 0.65-μm pore size filters to exclude larger bacterial cells and the obtained filtrates were subsequently spread on nutrient broth-soytone-yeast extract (NSY) (Hahn et al. 2004) agar plates and incubated at room temperature (about 21 °C). The agar plates were observed for several weeks. Growing colonies with the typical red pigmentation of strains belonging to the genus *Aquirufa* were picked and transferred to 24 well plates filled with liquid NSY medium. Promising cultures showing the expected pigmentation were transferred to Erlenmeyer flasks and purified by alternating culturing in a liquid medium and on agar plates. Because the 16S rRNA gene is in the case of the genus *Aquirufa* not suitable to distinguish between species (Pitt et al. 2024), we used as a marker the partial sequences of the *gyrB* gene encoding for the B subunit of the DNA gyrase (Pitt et al. 2022) and a threshold of 97% to assign the strains to a known species (Pitt et al., in revision). In addition, the strains which likely represent a new species were genome-sequenced and gANI values were calculated. All strains were stored at − 80 °C in a liquid NSY medium supplemented with 15% glycerol.

### Whole-genome sequencing and analyses

DNA extraction and genome sequencing were performed following the method described previously (Hoetzinger et al. 2017). A shotgun library was paired-end sequenced on an Illumina NovaSeq sequencer with 2 × 150 bp. For the de novo assembly of the genomes served SPAdes version 3.13.1 (Bankevich et al. 2012). The three genomes described here were deposited in DDBJ/ENA/GenBank databases and annotated by the NCBI Prokaryotic Genome Annotation Pipeline (Tatusova et al. 2016). In addition, the genomes were annotated by the Integrated Microbial Genomes & Microbiomes Expert Review (IMG/MER) system (Chen et al. 2019) and added to the IMG/MER database. The IMG/MER system served also for calculating gANI values and corresponding alignment fraction (AF) values for all possible pairwise comparisons of the new strains with type strains of previously established *Aquirufa* species. Digital DNA-DNA hybridization (dDDH) values were calculated in addition using the Type (Strain) Genome Server (Meier-Kolthoff et al. 2021) for the new strains and closely related type strains. For visualization of the genome sequences, we used the tools Proksee (Grant et al. 2023), BLAST (Altschul et al. 1990) and Alien Hunter (Vernikos and Parkhill 2006). Besides the genome sequences of the three strains described in this study, we used 36 further previously established genome sequences of *Aquirufa* strains (Table S1), which were almost exclusively isolated from freshwater habitats in Austria, for calculating intra-species gANI and AF values with the IMG/MER system. For a graphical representation of the values served R (RCoreTeam 2020) and RStudio (RStudioTeam 2019).

### Phylogenetic reconstructions

For phylogenetic reconstructions, we considered besides all type strains of the genus *Aquirufa*, type strains of the closely related genera *Sandaracinomonas*, *Pseudarcicella*, *Arcicella*, and *Flectobacillus* with available genome sequences.

A phylogenetic tree based on almost full-length 16S rRNA gene sequences was calculated with the software Mega X (Kumar et al. 2018). All sequences were aligned, trimmed, and utilized for the construction of a neighbor-joining tree with 1000 replicates based on the Kimura 2 parameter model (Kimura 1980) with the settings gamma-distributed (1 gamma Parameter) and gaps pairwise deleted. A genome-based phylogenetic tree was calculated by the use of amino acid sequences of the core genes. Therefore, all used genome sequences were annotated by Prokka version 1.14.6 (Seemann 2014) with the standard settings. The core genes of the sequence set were identified with the tool Roary version 3.13.0 (Page et al. 2015), with the setting of 65% minimum protein sequence similarity and the requirement that a gene/protein has to occur in all genomes. For both tools, we utilized The European Galaxy server (Galaxy-Community 2024). The obtained sequence set of 658 core genes was translated into protein sequences with Mega X (Kumar et al. 2018) and aligned by MAFFT (Katoh et al. 2005). The tool GBLOCKS, version 0.91b (Castresana 2000) served to filter out highly variable positions, which reduced the primary alignment from 247,753 to 228,366 alignment positions (92%) in 1164 selected blocks. A RAxML tree (Stamatakis 2014) was constructed on the platform CIPRES Science Gateway version 3.3 (Miller et al. 2010), with standard settings and 100 bootstrap replicates.

### Metagenome sequencing and analyses

Metagenome sequencing was performed with the water sample of the Ausee from November 2023. Approximately 300 ml of sampled water was prefiltered through 0.8-μm pore size filters to exclude larger particles and large bacterial cells and to enrich smaller ones like the target bacteria. The filtrate was subsequently filtered onto 0.2-μm pore size filters. DNA was extracted using phenol–chloroform-isoamyl alcohol as reported by Schauer et al. (2003). The library was sequenced on an Illumina NovaSeq with sequence mode NovaSeq PE 150, which resulted in 8.4 Gbp in 28.2 million reads. The metagenome as well as 123 publicly available metagenomes from freshwater habitats obtained from locations all over the world were analyzed by mapping as follows: We prepared a set of concatenated genome sequences with removed ribosomal operons of all *Aquirufa* type strains including 1-SAACH-A3^T^ and 2-BAHN-186B^T^. On the European Galaxy server (Galaxy-Community 2024), the reads of the metagenomes were mapped on the genome set with Bowtie2 (Langmead and Salzberg 2012) with settings selectively mapping only reads with whole-length (option end-to-end) and sequence identities of ≥ 95% reflecting the species demarcation threshold. Subsequently, the coverage for every base of the genome set was obtained by the use of the software Bedtools (Quinlan and Hall 2010). The data were analyzed with R (RCoreTeam 2020) and RStudio (RStudioTeam 2019). Infrequent occurring species could cause results characterized by low coverage breadth combined with low coverage depth, potentially indicating that the number of sequenced base pairs limited the detection of the reference genome. So, we used the following analysis to create a test criterion for distinguishing between detection and no detection. Metagenomes harboring genomes of *Polynucleobacter paneuropaeus* (i.e., mapping results with coverage depth > 100-fold and breadth > 70%) were separately mapped on genome sequences of five *Polynucleobacter paneuropaeus* strains (ANI > 98% but differing in their sets of accessory genes). The number of mapped metagenomic reads was stepwise reduced and the respective coverage depth and breadth were recorded. The plotted results showed for all five *Polynucleobacter paneuropaeus* genomes asymptotic curves (Fig. S1). The genome-specific data set with the smallest asymptote (reflecting the size of the core genome) was used to model the curve (Fig. S1). The obtained function (Fig. S1) was used to test whether a certain mapping result must be considered as detection. If the coverage breadth obtained by mapping metagenomic reads is at least tenfold and larger than the calculated coverage breadth (Fig. S1), a particular species is supposed to be detected. The obtained metagenomic reads from the Ausee sample were assembled by the use of metaSPAdes 3.15.3 (Nurk et al. 2017) and merged into metagenome-assembled genomes (MAGs) with MetaBat2 (Kang et al. 2019). CheckM lineage_wf (Parks et al. 2015) and GTDB-Tk Classify genomes (Chaumeil et al. 2019) were used for quality check and taxonomic classification, respectively. All tools were utilized on the European Galaxy server (Galaxy-Community 2024). GANI values were calculated with the ANI Calculator on Ezbiocloud (Yoon et al. 2017).

### Phenotypic and chemotypic characterization

Three of the obtained strains were phenotypically and chemotypically characterized with the same set of investigations (Pitt et al. 2024) and the same methods (Pitt et al. 2022) as reported previously. In brief, potential anaerobic growth, the temperature range of growth, and tolerance for NaCl were tested in each case on NSY agar plates inoculated with a well-growing liquid culture. For testing anaerobic growth, an anaerobic jar (oxygen removed with crystalline silicic acid) and in addition to standard NSY plates, NSY plates supplemented with 2 g l^−1^ NaNO_3_ were used to specifically test for nitrate respiration. Temperatures were tested starting at 5 °C and increased if the strains showed growth. Close to the supposed tolerated temperature, we used 1 °C steps until the strains showed no growth. The same procedure served for testing NaCl tolerance by using steps of 0.1% w/v. For testing the motility of the strains, soft agar plates (0.1 g l^−1^ K_2_HPO_4_, 1 g l^−1^ yeast extract, and 2.0 g l^−1^ agar) were inoculated with a drop of culture and monitored for fourteen days. Spreading over the whole agar plate within 2 weeks was regarded as a positive result. Cell shapes and metrics were determined with an epifluorescence microscope (UV filter) after fixing liquid cultures with 2% paraformaldehyde and staining by 4′,6-diamidino-2-phenylindole (DAPI). The composition of the fatty acids, polar lipids, and respiratory quinones were determined. For these purposes, the biomass of liquid cultures (NSY medium, room temperature, 3-day incubation) was harvested by centrifugation. After saponification and methylation, the fatty acid content was analyzed by gas chromatography coupled to a flame ionization detector as described previously (Sasser 1990). The identity of fatty acid species was determined by gas chromatography/mass spectrometry (GC/MS) (Vieira et al. 2021). Double bond-containing species were further resolved by derivatization to dimethyl disulfide adducts (Moss and Lambert-Fair 1989) and subsequent GC/MS analysis. The polar lipids were extracted and analyzed by thin-layer chromatography according to the methods of Tindall (Tindall 1990a, 1990b), which was based on the description by Bligh and Dyer (Bligh and Dyer 1959). Total lipids were detected by spraying the plates with dodecamolybdophosphoric acid (Dmp). Specific functional groups were visualized with α-naphthol, ninhydrin, and molybdenum blue. Respiratory quinones were extracted via solid-phase extraction and analyzed by reversed-phase HPLC coupled to a diode array dector as described previously (Vieira et al. 2021).

## Results and discussion

### Habitats

An overview of the restoration area and the location of the sampling sites is given in Fig. 1, and all data concerning the sampled habitats are shown in Table 1. The site at the river Salzach was characterized by electrical conductivity values between approximately 200 μS/cm and 350 μS/cm and pH values between 7 and 8.5; the water temperatures varied from 5.8 °C in winter to 16.4 °C during the summer sampling (Table 1). The data of the nearby creek Reitbach showed similar magnitudes but higher fluctuations (Table 1). The fluctuations were even higher at the two sampling sites with standing waters, so the lake Ausee and the newly created small pond. They reached high temperatures of about 25 °C in summer and low temperatures of around 4 °C in winter. Probably due to high primary productivity, the pH value raised in summer in the case of the Ausee to almost 9. The saturation of oxygen was in all four habitats during the sampling period between 80 and 100%; therefore, enough oxygen for aerobic bacteria was available in the upper water layers (sampling at 0.1 m depth).

**Table 1:** Characteristics of the four habitats of the investigated area and taxonomic assignment of the isolated strains. Messurements were performed in 0.1-m depth. 1, Salzach; 2, newly created pond; 3, Ausee; 4, Reitbach

	1	2	3	4
Habitat type	River	Small pond	Lake	Creek
Geographic coordinates	47.914518 N 12.954952 E	47.923345 N 12.962754 E	47.920563 N 12.959348 E	47.914978 N 12.956025 E
Width or area	158 m	0.16 ha	9.99 ha	9 m
Sampling 12.07.2023				
pH	8.5	8.9	8.1	n.d.*
Electrical conductivity	214 μS/cm	174 μS/cm	370 μS/cm	n.d.*
Water temperature	16.4 °C	25.9 °C	25.3 °C	n.d.*
Oxygen	9.1 mg/L, 96.3%	7.8 mg/L, 99.6%	7.7 mg/L, 97.8%	n.d.*
Isolated strains belong to	***A. salirivi*** *A. ecclesiirivi* *A. antheringensis*	No cultures obtained	No cultures obtained	No culturing experiment
Sampling 8.11.2023				
pH	7.2	6.7	6.8	8.1
Electrical conductivity	297 μS/cm	265 μS/cm	350 μS/cm	455 μS/cm
Water temperature	9.3 °C	8.5 °C	10.4 °C	8.6 °C
Oxygen	9.15 mg/L, 83.5%	9.66 mg/L, 86.1%	8.8 mg/L, 81.7%	9.33 mg/L, 84.4%
Isolated strains belong to	*A. antheringensis*	***A. novilacunae***	*A. antheringensis* ***A. novilacunae*** *A. ecclesiirivi* *A. regiilacus* *A. aurantiipilula* *A.* sp.	*A. antheringensis* *A. nivalisilvae*
Sampling 31.1.2024				
pH	7.6	8.1	7.4	8.5
Electrical conductivity	348 μS/cm	296 μS/cm	360 μS/cm	468 μS/cm
Water temperature	5.8 °C	4.3 °C	4.8 °C	4.1 °C
Oxygen	10.4 mg/L, 86%	12.7 mg/L, 102.8%	9.5 mg/L, 80.1%	11.5 mg/L, 90.3%
Isolated strains belong to	No culturing experiment	No culturing experiment	No culturing experiment	No culturing experiment
Sampling 16.04.2024				
pH	7.4	7.9	7.8	6.7
Electrical conductivity	196 μS/cm	352 μS/cm	350 μS/cm	233 μS/cm
Water temperature	15 °C	14.3 °C	14.2 °C	9.6 °C
Oxygen	n.d	9.0 mg/L, 91.9%	10.3 mg/L, 104.8%	10.5 mg/L, 97.2%
Isolated strains belong to	No cultures obtained	No cultures obtained	No cultures obtained	No cultures obtained

^*^Additional sampling 03.09.2024 pH 7.9, conductivity 406 μS/cm, temperature 19.4 °C, oxygen 83.9%

### Isolated strains and metagenome of the lake Ausee

The water samples taken in the summer and autumn of 2023 and the spring of 2024 were used to get bacterial strains belonging to the genus *Aquirufa* (Table 1). Interestingly, the sampling in summer resulted only in the case of the river Salzach in *Aquirufa* isolates. The obtained strains belonged to two known species, and one of them represented a new species (Table 1). While not a single *Aquirufa* isolate was received from the samplings in spring, we got pure cultures from all four water samples in autumn (Table 1). Numerous strains originated from the lake Ausee, they belonged to six different species, including two so far undescribed ones (Table 1). For further analyses aiming to describe new *Aquirufa* species, we selected strain 1-SAACH-A3^T^ originating from the river Salzach, and 2-BAHN-186B^T^ and AUSEE-184A6 originating from the newly created pond and the lake Ausee, respectively.

The analyses of the metagenome sequence of the autumn sample of the Ausee could not confirm the findings of the cultivation experiment. Mapping of the obtained metagenomic reads on *Aquirufa* genomes resulted only in the detection of *A. antheringensis* (coverage breadth 60.3% of the genome, 3.1 reads/covered position). The metagenome assembly and binning resulted in a metagenome-assembled genome (MAG) with 0.7 Mbp (276 contigs) with 28.2% completeness, 0.86% contamination, but 100% heterogeneity. The taxonomic assignment revealed that it should belong to unknown *Aquirufa* species. Calculation of gANI value with *Aquirufa* strains resulted in 89.7% for the type strain of *A. antheringensis* as the highest value, with a coverage of 68.6%. Thus, the MAG could not be assigned to a previously described species or a new species discovered through the cultivation experiments, likely it consisted of small parts of two or more unknown *Aquirufa* species. Finally, BLAST comparisons with *gyrB* gene sequences of genome-sequenced *Aquirufa* strains against the assembled metagenomic contigs resulted in the detection of *A. antheringensis* (identity 97%) and strain 2-AUSEE-184A6 (identity 99% and 100%, respectively). All these results suggested that *Aquirufa* populations were not abundant enough in the lake Ausee sample to get precise information about the species composition through metagenome sequencing.

### Strains representing new species

#### Phenotypic and chemotypic characteristics

The phenotypic and chemotypic characteristics of the three strains, 1-SAACH-A3^T^, 2-BAHN-186B^T^, and 2-AUSEE-184A6 are shown in Table 2, the fatty acid compositions in Table S2, and the patterns of the polar lipids in Fig. S2. All characteristics of these strains resembled the characteristics of the related type strains, only slight differences concerning the respiratory quinones, the fatty acids, and polar lipids could be observed (Table 2).

**Table 2:** Features of the new strains and the type strains of the nearest related species

	**1**	2	3	**4**	**5**	6	7
Mean cell length (μm)	1.2	1.7	1.5	1.2	1.4	1.7	1.2
Mean cell width (μm)	0.4	0.5	0.3	0.4	0.6	0.6	0.5
Temperature range for growth (°C)	5–32 (w)	5–34	5–30 (w)	5–32 (w)	5–32 (w)	5–32 (w)	5–31 (w)
NaCl tolerance (%, w/v)	0–0.2	0–0.4	0–0.2 (w)	0–0.1	0–0.1	0–0.3 (w)	0–0.1 (w)
*Respiratory quinones* (% of the total content):							
Menaquinone-7	96.7	100.0	≥ 99.0	94.7	95.5	≥ 99.0	99.4
Menaquinone-6	3.1	-	< 1.0	3.4	3.2	< 1.0	-
Menaquinone-8	0.2	-	-	1.9	1.3	-	0.6
*Major fatty acids* (% of the total content):							
C_16:1_ω5c	9.5	13.9	6.7	10.1	8.1	11.9	7.0
C_16:1_ω7c	23.1	21.0*	21.0*	12.1	6.9	24.7*	12.0
iso-C_15:0_	21.8	24.5	22.7	34.9	48.0	20.3	41.4
anteiso-C_15:0_	8.8	8.4	8.3	11.3	13.2	5.6	15.5
iso-C_15:0_3-OH	10.8	9.3	8.3	9.4	4.7	13.4	4.5
*Polar lipids:*							
Unidentified phospholipids	1	-	1	1	-	-	-
Unidentified aminolipid	-	-	1	-	-	-	-
Unidentified aminophospholipids	3	3	3	3	2	2	3
Unidentified glycolipids	2	-	-	3	2	-	-
Unidentified polar lipids	4	2	2	3	3	4	2

**1, 1-SAACH-A3**^**T**^; 2, *A. ecclesiirivi* 50A-KIRBA^T^; 3, *A. beregesia* 50C-KIRBA^T^; 4, **2-BAHN-186B**^**T**^; 5, **2-AUSEE-184A6**; 6, *A. antheringensis* 30S-ANTBAC^T^;** 7**, *A lenticrescens* 9H-EGSE^T^

All strains had in common: cell morphology: rods, pigmentation of colonies: red, pigmentation in liquid medium: red-orange, anaerobic growth: -; gliding on soft agar: + ; identified polar lipid: phosphatidylethanolamine. Only the major fatty acids (≥ 10% for at least one strain) are listed, the whole fatty acid composition of the new strains is shown in Table S2. *, and/or iso-C_15:0_2-OH; − , negative; + , positive; w, weak

All data were elevated in the same laboratories under the same conditions, data from columns 2, 3, 6, and 7 were published earlier (Pitt et al. 2019, 2020, 2022, 2024)

#### Phylogenetic position

**Fig. 2:**
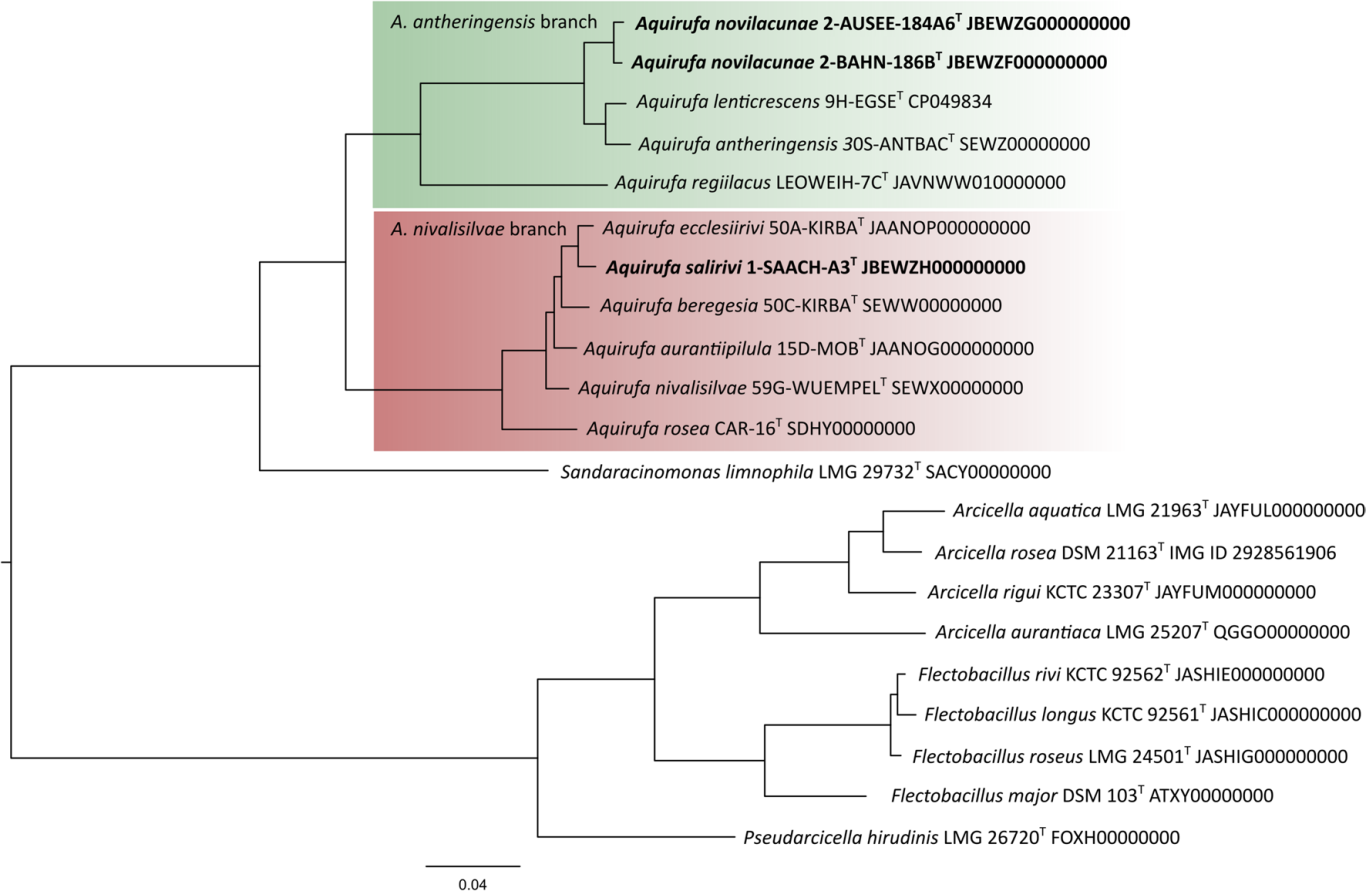
Phylogenetic reconstruction of the new strains, all *Aquirufa* type strains and type strains of closely related genera. Shown is a midpoint-rooted RaxML tree based on the amino acid sequences of 658 core genes. Bootstrap values for all nodes 100%

Phylogenetic reconstructions with 16S rRNA gene sequences placed strain 1-SAACH-A3^T^ on the *A. nivalisilvae* branch of the genus *Aquirufa*; 2-BAHN-186B^T^ and 2-AUSEE-184A6 were situated on the *A. antheringensis* branch (Fig. S3). BLAST searches with the NCBI database (Johnson et al. 2008) revealed 16S rRNA gene sequence similarity values for strain 1-SAACH-A3^T^ of 99.9% compared with nearly all species of the branch (*A. rosea* 99.5%). The two other new strains had identical 16S rRNA gene sequences, the highest similarity values amounted to 99.9% and 99.8% for comparison with *A. lenticrescens* and *A. antheringensis*, respectively*.* The genome-based phylogenetic tree calculated by using amino acid sequences of 658 core genes (Fig. 2) showed a more differentiated insight into the phylogeny of the new strains. While the allocation of the new strains to the two *Aquirufa* branches was reflected as well, 1-SAACH-A3^T^ was very close to *A. ecclesiirivi*, while 2-BAHN-186B^T^ and 2-AUSEE-184A6 were on a separated branch beside *Aquirufa lenticrescens* and *Aquirufa antheringensis* (Fig. 2). Overall, the genome-based tree revealed the phylogenetic distances between the regarded strains, which could not be recognized in the 16S rRNA gene-based tree.

#### Genomic characteristics

The authenticity of all assembled genomes could be confirmed by comparison of the 16S rRNA gene Sanger sequences with the genome sequences. The genome of strain 1-SAACH-A3^T^ consisted of 56 contigs, had a nucleotide coverage of 403 × , a size of 3.2 Mbp and a G + C content of 38.4 mol%. The strain fit regarding the genome size and a G + C content of 38.4% very well to the related species of the *A. antheringensis* branch (Table 3). Like the type strains of *A. ecclesiirivi* and *A. beregesia*, the genome of 1-SAACH-A3^T^ included an operon for the assimilatory usage of nitrate and nitrite including genes predicted for nitrate reductase, nitrite reductase, a nitrate/nitrite MFS transporter, and rubredoxin. The genome contained also an operon encoding genes for the reduction of nitrous oxide to molecular nitrogen including genes predicted for nitrous oxide reductase and cytochrome c551/c552 (Table 3). The assembled genome of strain 2-BAHN-186B^T^ consisted of 6 contigs, had a nucleotide coverage of 762 × , a size of 2.4 Mbp and a G + C content of 42.4 mol%, of strain 2-AUSEE-184A6 of 7 contigs, coverage 1423 × , a size of 2.4 Mbp and a G + C content of 42.2 mol%. They matched very well with the species of the *A. antheringensis* branch regarding the genome size, G + C content, typical high coding density, and the number of protein-coding genes (Table 3). Like the type strains of *A. antheringensis* and *A. lenticrescens*, the genomes of the two strains encoded genes predicted for the light-harvesting rhodopsin system (Table 3).

**Table 3:** Genomic comparison of the new strains and type strains of related species

	1	2	3	4	5	6	7
IMG/MER ID number	807,111763	2828879446	2816332124	8103260213	8099168989	2816332120	2857132225
Genome size (Mbp)	3.2	3.1	3.2	2.4	2.4	2.5	2.5
DNA G + C (mol%)	38.4	38.4	38.5	42.4	42.2	42.6	42.2
Coding density	92.5	92.3	92.2	95.0	95.1	94.8	95.0
Protein coding genes	2742	2663	2743	2158	2168	2224	2218
gANI with 1-SAACH-A3^T^ (%)	100	93.65	88.28	75.43	75.41	75.41	75.45
Corresponding average AF (%)	100	90.8	86.41	41.24	41.0	40.18	39.92
gANI with 2-BAHN-186B^T^ (%)	75.43	75.37	75.38	100	95.76	82.99	82.97
Corresponding average AF (%)	32.2	32.39	31.19	100	93.82	85.46	84.8
dDDH with 1-SAACH-A3^T^ (d_4_, %)	100	51.4	34.7	18.4	18.3	19.0	18.5
dDDH with 2-BAHN-186B^T^ (d_4_, %)	18.4	18.1	18.2	100	65.5	60.5	60.7
Genes putatively encoding for:							
Bacteriorhodopsin COG5524	-	-	-	+	+	+	+
Beta-carotene 15,15’-dioxygenase EC:1.13.11.63	-	-	-	+	+	+	+
Nitrous oxide reductase EC:1.7.2.4	+	+	+	-	-	-	-
Nitrate reductase (assimilatory) EC:1.7.7.2	+	+	+	-	-	+	-
Nitrite reductase (assimilatory) EC:1.7.1.15	+	+	+	-	-	+	-
Nitrate/nitrite transporter COG2223	+	+	+	-	-	+	-

**1, 1-SAACH-A3**^**T**^; 2, *A. ecclesiirivi* 50A-KIRBA^T^; 3, *A. beregesia* 50C-KIRBA^T^; 4, **2-BAHN-186B**^**T**^; 5, **2-AUSEE-184A6**; 6, *A. antheringensis* 30S-ANTBAC^T^;** 7**, *A lenticrescens* 9H-EGSE^T^

#### Ecology and distribution

Like the related type strains of the *A. nivalisilvae* branch (Table 3), 1-SAACH-A3^T^ showed properties which could be interpreted as a combination of pelagic lifestyle and association with particles or surfaces (Chiriac et al. 2023) such as small cell sizes, intermediate genome sizes, and genes for uptake of nitrate or utilization of nitrous oxide, which occurs at the aerobic/anaerobic layer. Interestingly, neither further cultivation efforts in Austria (Salzburg and Upper Austria) (unpublished data) nor the analysis of 123 publicly available metagenomes indicated the occurrence of the new species represented by 1-SAACH-A3^T^ in other freshwaters. So, it seemed that the strain represents a rare species.

In contrast, strains 2-BAHN-186B^T^ and 2-AUSEE-184A6 showed characteristic properties of freshwater bacteria with a pelagic lifestyle (Chiriac et al. 2023) such as small cell sizes, comparatively low genome sizes, high coding densities of the genomes, and light-harvesting systems, like the related type strains of the *A. antheringensis* branch (Table 3). Culturing experiments with freshwater samples derived from various sites in Austria (Salzburg and Upper Austria) resulted in no further strains belonging to the species represented by 2-BAHN-186B^T^ and 2-AUSEE-184A6 (unpublished data). Nevertheless, the investigation of 123 publicly available metagenomes from freshwater habitats resulted in 15 cases in detections of the new species represented by the two strains (Table S3). While evidence for the new species was exclusively found in rivers, most of these habitats were located in Asia, the River Geum, The Republic of Korea, and the Jinsha and Yangtze Rivers in China. Two detections were also made in water samples of the Columbia River Estuary, USA, and the Torrens River, Australia (Table S3). It was difficult to quantify the relative abundances of the new species based on the metagenomic sequences, especially because freshwater bacteria have a wide range of genome sizes, which can lead to an over- or underestimation. Since the new species has an average genome size considering planktonic freshwater bacteria, the percentage of metagenomic reads mapping on the genome of strain BAHN-186B^T^ (Table S3) could give insights into the relative abundances. According to these data the highest abundances of the new species were detected in water samples from the Yangtze River (SRR9924798 and SRR9924799, Table S3) with 0.188% and 0.106% mapped reads, respectively. Both habitats were situated in the subtropical climate zone.

The data of the metagenome analyses and the data of the home habitats of the two strains representing the new species suggested that it occurs in standing and running freshwaters as well, maybe in populations with adaptations to the habitat conditions through different genotypes.

## Conclusions and evidence for two new species

Concerning the culturing experiments, the greatest biodiversity of *Aquirufa* species was found in the lake Ausee with six distinct species, while water samples from the river Salzach, the creek Reitbach, and the small recently established pond resulted only in cultures of three, two, and one species, respectively. It is necessary to consider that certainly not all of the present species could be detected by culturing experiments. Since metagenomic sequencing of the lake Ausee sample was not appropriate to get insights into the biodiversity of the genus *Aquirufa*, further methods are needed. Nevertheless, the results showed some interesting points. First, it seemed that the lake Ausee served as a habitat for a larger number of *Aquirufa* species; maybe, it acts as a reservoir for the populations to colonize the surrounding water bodies. Second, the occurrence of *Aquirufa* strains in the upper water layer seems to undergo seasonal fluctuations, with a peak in autumn.

**Fig. 3:**
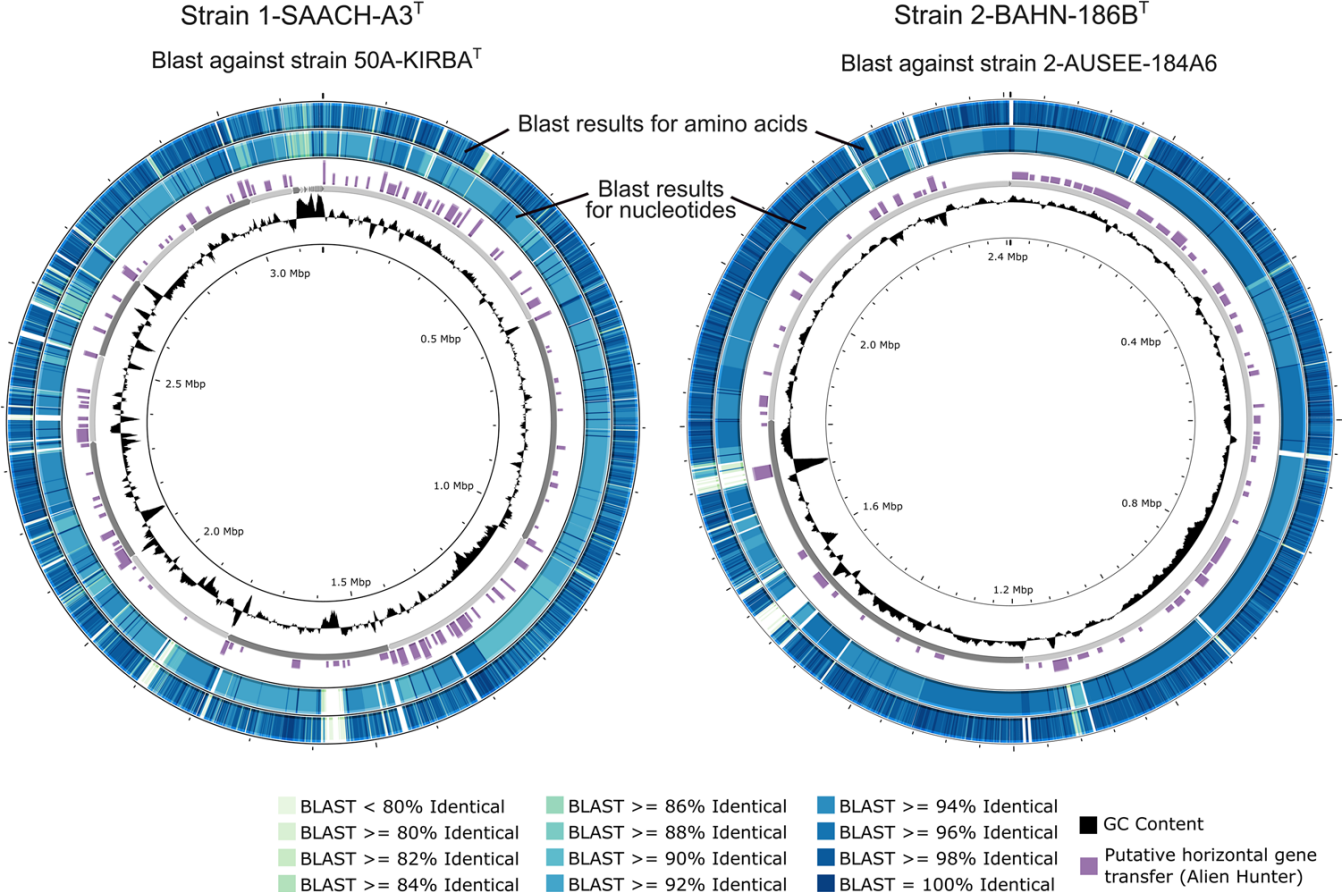
Visualization of the genomes of strains 1-SAACH-A3^T^ and 2-BAHN-186B^T^. Shown is the G + C-content (peaks indicate values higher or lower than the average G + C content), score of putative horizontal gene transfer and comparison with strain 50A-KIRBA.^T^ and strain 2-AUSEE-184-A6, respectively as BLAST results for nucleotides (inner blue circle) and BLAST results for amino acids (outer blue circle)

**Fig. 4:**
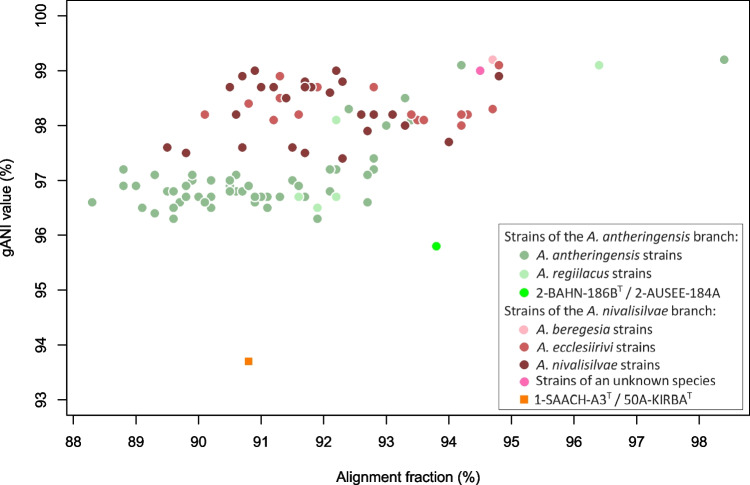
Intra-species gANI (whole genome average nucleotide identity) values and alignment fraction values of strains of the genus *Aquirufa* (circle points) and inter-species values of the new strain 1-SAACH-A3^T^ compared with its closest known relative *A. ecclesiirivi* strain 50A-KIRBA.^T^ (square point). The colors of the points reflect the assignment to a particular species and to one of the clusters *A. antheringensis* branch and *A. nivalisilvae* branch, respectively (see legend)

Of special interest were the three new strains, which represent new species. Table 3 indicates the overall genome-related indexes gANI and dDDH of the new strains and the nearest related type strains of *Aquirufa* species. In the case of 1-SAACH-A3^T^, all gANI values were below the proposed threshold of 95% (Konstantinidis et al. 2006; Jain et al. 2018) but in the case of the type strain of *Aquirufa ecclesiirivi* with 93.65%, close to the threshold value. Calculations of dDDH values with the same genome set (Table 3) resulted in a maximum value of 51.4% and were more significantly below the used threshold of 70% (Chun et al. 2018). The calculated gANI value comparing strains 2-BAHN-186B^T^ and AUSEE-184A6 of 95.76% was also unusual because it was very close but above the proposed threshold. Comparisons with the closest related type strains of *Aquirufa* species revealed gANI values around 83%, which indicated clearly that they represent at least one new species. Determination of the dDDH value comparing 2-BAHN-186B^T^ and AUSEE-184A6 resulted in 65.5%, a value slightly below the proposed threshold of species separation. So, more evidence was needed for the assumption that strain 1-SAACH-A3^T^ represented a new species and that strains 2-BAHN-186B^T^ and AUSEE-184A6 belonged to the same species. Figure 3 shows BLAST comparisons of the latter two strains and the pair 1-SAACH-A3^T^/50A-KIRBA^T^ (type strain of *A. ecclesiirivi*). The figure displayed in the case of 1-SAACH-A3^T^/50A-KIRBA^T^ (Fig. 3, left side) regions with comparatively low identity values for nucleotides and for amino acids, the comparison of strains 2-BAHN-186B^T^ and AUSEE-184A6 yielded a more uniform pattern (Fig. 3, right side). Since calculating gANI values is the most used and accepted method for species demarcation, we focused on deeper insights into the prevalence. As strain 1-SAACH-A3^T^ belongs to the *A. nivalisilvae* branch of the genus *Aquirufa* and 2-BAHN-186B^T^ and AUSEE-184A6 to the *A. antheringensis* branch, we checked if there are any differences concerning the intra-species gANI values and corresponding AF values between the two groups. Figure 4 shows a plot of 107 intra-species gANI/AF value pairs derived from genome sequences of strains belonging to *Aquirufa* species (Table S1); in addition, strains 2-BAHN-186B^T^ compared with AUSEE-184A6 (bright green dot) and 1-SAACH-A3^T^ compared with 50A-KIRBA^T^ (orange dot). The figure showed an interesting pattern. On the one hand, strains belonging to the *A. antheringensis* branch were characterized by comparably low intra-species gANI values and strains of the *A. nivalisilvae* branch by higher gANI values (Fig. 4). If we assume that strain 1-SAACH-A3^T^ represents a new species and strains, 2-BAHN-186B^T^ and AUSEE-184A6 represent together a second new species; the three new strains fit very well into the picture. A model by Hoetzinger et al. (2024) tries to explain different scales in intra-species ANI values. Accordingly, species with comparatively low ANI values consist of bigger and more stable populations which are hardly connected by gene flow. On the other hand, species with higher ANI values consist of small fluctuating populations with high gene flow (Hoetzinger et al. 2024). The observed higher horizontal gene transfer for strain 1-SAACH-A3^T^ compared with strain 2-BAHN-186B^T^ (Fig. 3) fit the model well. While 1-SAACH-A3^T^ was the only received strain belonging to the new species, several strains which could be assigned to the other new species by sequencing of the *gyrB* gene were cultured from the water samples of the lake Ausee and the newly created pond (data not shown). This could be a hint that the population sizes were here larger. In addition, the genome-based phylogenetic tree (Fig. 2) confirmed the assumption that the three new strains represent two new species. The branch lengths, representing the phylogenetic distance between the strains 2-BAHN-186B^T^ and 2-AUSEE-184A6, were comparably small; however, the phylogenetic distance between 1-SAACH-A3^T^ and the nearest relative *A. ecclesiirivi* was in the same range as between other *Aquirufa* species or species of the genus *Flectobacillus* (Fig. 2). As mentioned above, further investigations are necessary to explore the biodiversity and distribution of the genus *Aquirufa* more precisely. In this study, we propose the establishment of two new species, *Aquirufa salirivi* sp. nov. with 1-SAACH-A3^T^ as type strain and *Aquirufa novilacunae* sp. nov. with 2-BAHN-186B^T^ as type strain.

### Description of *Aquirufa salirivi* sp. nov.

*Aquirufa salirivi* (sa.li.ri’vi. L. masc. n. *sal*, salt; L. masc. n. *rivus*, river; N.L. gen. n. *salirivi*, of a salt river, referring to the river Salzach, named after transporting salt on it).

Cells of the type strain form rods with an approximate size of 1.2 × 0.4 μm. Liquid cultures have a red–orange coloring, and colonies on agar plates are red-pigmented. Cultures show motility on soft agar plates and grow at 5–32 °C and in 0–0.2% (w/v) NaCl. Major fatty acids are C_16:1_ω7c, iso-C_15:0_, and iso-C_15:0_3-OH. Polar lipids are phosphatidylethanolamine, unidentified phospholipids, unidentified aminophospholipids, unidentified glycolipids, and further unidentified lipids. Menaquinone-7 occurs as a major respiratory quinone, small amounts of menaquinone-6 and traces of menaquinone-8 are present. The genomic DNA of the type strain has 3.2 Mbp and a G + C content of 38.4 mol%.

The type strain is 1-SAACH-A3^T^ (= DSM 117800^ T^ = JCM 37097^ T^), isolated from water of the river Salzach (Salzburg, Austria). Sequence accession numbers: 16S rRNA gene PQ394666, whole genome JBEWZH000000000.

### Description of *Aquirufa novilacunae* sp. nov.

*Aquirufa novilacunae* (no.vi.la.cu’nae. L. masc. adj. *novus*, new; L. fem. n. *lacuna*, pond; N.L. gen. n. *novilacunae*, of a new pond).

Cells form rods with an approximate size of 1.3 × 0.5 μm. Liquid cultures have a red–orange coloring, and colonies on agar plates are red-pigmented. Cultures show motility on soft agar plates and grow at 5–32 °C and in 0–0.1% (w/v) NaCl. Major fatty acids are iso-C_15:0_, C_16:1_ω7c, anteiso-C_15:0_, C_16:1_ω5c, and C_16:1_ω7c. Polar lipids are phosphatidylethanolamine, unidentified phospholipids, unidentified aminophospholipids, unidentified glycolipids, and further unidentified lipids. Menaquinone-7 occurs as a major respiratory quinone, small amounts of menaquinone-6 and menaquinone-8 are present. The genomic DNA of the type strain has 2.4 Mbp and a G + C content of 42.4 mol%.

The type strain is 2-BAHN-186B^T^ (= DSM 118143^ T^ = JCM 37099^ T^), isolated from the water of a small pond in Weitwörth (Salzburg, Austria). Sequence accession numbers: 16S rRNA gene PQ397515, whole genome JBEWZF000000000. Strain AUSEE-184A6 (= DSM 117801 = JCM 37098), isolated from water of the lake Ausee in Weitwörth (Salzburg, Austria) belongs also to the species. Sequence accession numbers: 16S rRNA gene PQ394667 accession number whole-genome sequence: JBEWZG000000000.

## Supplementary Information

Below is the link to the electronic supplementary material.

Supplementary file1 (PDF 633 KB)

## Data Availability

The Whole Genome Shotgun project has been deposited at DDBJ/ENA/GenBank under the accession JBEWZH000000000 for strain 1-SAACH-A3T, JBEWZF000000000 for strain 2-BAHN-186BT and JBEWZG000000000 for strain 2-AUSEE-184A6. These are the versions described in this paper. The accession of the 16S rRNA gene sequence deposited at DDBJ/ENA/GenBank is PQ394666 of strain 1-SAACH-A3T, PQ397515 of strain 2-BAHN-186BT, and PQ394667 of strain 2-AUSEE-184A6.

## Abbreviations

*A.*: *Aquirufa*
gANI: Whole-genome average nucleotide identity
NSY medium: Nutrient broth-soytone-yeast extract medium
*gyrB*: B subunit of the DNA gyrase
IMG/MER: Integrated Microbial Genomes & Microbiomes Expert Review
AF: Alignment fraction
dDDH: Digital DNA-DNA hybridization
MAG: Metagenome-assembled genome
GC/MS: Gas chromatography/mass spectrometry
