# Supplementary material for: Biodiversity of strains belonging to the freshwater genus Aquirufa in a riparian forest restoration area in Salzburg, Austria, with a focus on the description of Aquirufa salirivi sp. nov. and Aquirufa novilacunae sp. nov
Source: Int Microbiol. 2025 Feb 18;28(7):1635–47. doi: 10.1007/s10123-025-00642-x (PMC12528234; doi:10.1007/s10123-025-00642-x)
Supplement: Supplementary file 1 — Supplementary file1 (PDF 633 KB) [file 10123_2025_642_MOESM1_ESM.pdf]

Supplementary information

Biodiversity of strains belonging to the freshwater genus *Aquirufa* in a riparian forest restoration area in Salzburg, Austria with a focus on the description of *Aquirufa salirivi* sp. nov. and *Aquirufa novilacunae* sp. nov.

Alexandra Pitt, Stefan Lienbacher, Johanna Schmidt, Meina Neumann-Schaal, Jacqueline Wolf, Hannah Wenng, Aharon Oren, Zoe Huber and Martin W. Hahn

Correspondence: Alexandra Pitt, alexandra.pitt@uibk.ac.at

Table S1 List of the additional genomes of *Aquirufa* strains used for Fig.4

Strain name	Species	IMG/MER identity number
15A-MOB	*Aquirufa antheringensis*	2857116723
30S-ANTBAC^{T}	*Aquirufa antheringensis*	2816332120
4B-EUGB	*Aquirufa antheringensis*	2857119029
103A-SOEBACH	*Aquirufa antheringensis*	2816332126
134A-ALMKL	*Aquirufa antheringensis*	2857114421
127A-TCHWSR	*Aquirufa antheringensis*	2828877133
44A-TON	*Aquirufa antheringensis*	2857112171
INPA-49A	*Aquirufa antheringensis*	8076100290
DOPA-48B	*Aquirufa antheringensis*	8076102586
PLAD-142H	*Aquirufa antheringensis*	8076104833
FRTEICH-102D4	*Aquirufa antheringensis*	8076107191
LEPPI-3A	*Aquirufa regiilacus*	8014893578
LEOWEIH-7C^{T}	*Aquirufa regiilacus*	8023692520
HALL-GUE30-B3	*Aquirufa regiilacus*	8076146341
GRABEN-B1	*Aquirufa regiilacus*	8099173678
50C-KIRBA^{T}	*Aquirufa beregesia*	2816332124
BHBGOP-31H	*Aquirufa beregesia*	8069787654
50A-KIRBA^{T}	*Aquirufa ecclesiirivi*	2828879446
2-AUSEE-172A13	*Aquirufa ecclesiirivi*	8099181405
5A-MARBSE	*Aquirufa ecclesiirivi*	2857129497
11K-KIROB	*Aquirufa ecclesiirivi*	2857126801
BABACH-43C	*Aquirufa ecclesiirivi*	8015553863
15D-MOB^{T}	*Aquirufa aurantiipilula*	2857134496
WAEICH-18A	*Aquirufa aurantiipilula*	8014895999
VOEWA-120C1	*Aquirufa aurantiipilula*	8076124206
SAAGRA-154A	*Aquirufa aurantiipilula*	8076126856
4D-EUGB	*Aquirufa nivalisilvae*	2857124097
59G-WUEMPEL^{T}	*Aquirufa nivalisilvae*	2816332125
HME7025	*Aquirufa nivalisilvae*	2811994884
16I-TONSE	*Aquirufa nivalisilvae*	2857121315
WATS-35A	*Aquirufa nivalisilvae*	8015551193
DARE-47E	*Aquirufa nivalisilvae*	8069790498
GARBAGER-33B	*Aquirufa nivalisilvae*	8076129643
ROBBACH-80M	*Aquirufa nivalisilvae*	8076121439
WALL-65K1	*Aquirufa sp.*	2958958940
OSTEICH-129A	*Aquirufa sp.*	8069798001

Table S2 Fatty acid composition of strains 1-SAACH-A3^T, 2-BAHN-186B^T, and 2-AUSEE-184A6. Only fatty acids with values ≥ 1 % for at least one of the strains were listed. Major fatty acids (> 10 %) were marked with bold letters.

	1-SAACH-A3^T	2-BAHN-186B^T	2-AUSEE-184A6
$C_{15:1}\omega4c$ (Unknown 14.959)	3.5	3.1	5.8
$C_{15:1}\omega6c$	3.1	1.1	0.7
$C_{16:1}\omega5c$	9.5	**10.1**	8.1
$C_{16:1}\omega7c$	**23.1**	**12.1**	6.9
$C_{17:1}\omega6c$	3.3	1.2	0.7
iso-$C_{11:0}$	2.0	2.8	3.1
iso-$C_{15:0}$	**21.8**	**34.9**	**48.0**
anteiso-$C_{15:0}$	8.8	**11.3**	**13.2**
iso-$C_{17:1}\omega5c$	2.2	2.3	1.6
iso-$C_{17:1}\omega7c$	2.1	1.3	0.7
iso-$C_{15:0}$3-OH	**10.8**	9.4	4.7
iso-$C_{16:0}$3-OH	0.5	0.8	1.3
iso-$C_{17:0}$3-OH	2.5	1.8	0.9

Table S3 Publicly available metagenomes with detection of the new species represented by strain 2-BAHN-186B^T and 2-AUSEE-184A. The three last columns indicate the mapping results for the type strain.

Habitat	Latitude Longitude ASL	Sampling Date	pH	Cond. (µS/cm)	T (°C)	Accession number run	Size meta-genome (Gbp)	Reference	Reads per covered position	Cover-age breadth (%)	Mapped reads (%)
Columbia River Estuary, United States	46.2327 N 123.9168 W 0 m	October 2012	8.0	200	n.d.	SRR5468264	4.8	No reference	1.4	23.6	0.017
Columbia River Estuary, United States	46.2314 N 123.8828 W 0 m	July 2013	8.0	200	n.d.	SRR5468274	4.9	No reference	1.6	34.0	0.026
Torrens River, Australia	34.917864 S 138.588777 E 21 m	February 2021	7.3	413	23.8	SRR22711022	22.9	No reference	4.0	71.0	0.030
River Geum, Republic of Korea	36.461306 S 127.095889 E 8 m	2016	8.0	350	n.d.	SRR12487025	16.6	(Shim et al. 2018)	2.0	65.5	0.019
Jinsha River, China	26.88 N 99.98 E 1817 m	October 2014	8.2	488	n.d.	SRR9924778	16.8	(Liu et al. 2020) Environmental data: (Yang et al. 2020)	3.1	84.2	0.037
Jinsha River, China	26.569125 N 101.701627 E 988 m	October 2014	8.2	488	n.d	SRR9924781	17.4	(Liu et al. 2020) Environmental data: (Yang et al. 2020)	3.3	88.5	0.040
Yangtze River, China	28.77 N 104.65 E 259 m	October 2014	8.0	405	n.d.	SRR9924785	14.9	(Liu et al. 2020) Environmental data: (Lu et al. 2023)	2.6	81.1	0.034
Yangtze River, China	28.897665 N 105.551587 E 217 m	October 2014	7.1	410	n.d	SRR9924784	14.3	(Liu et al. 2020) Environmental data: (Lu et al. 2023)	4.7	93.7	0.074
Yangtze River, China	29.019678 N 105.85225 E 191 m	October 2014	7.8	410	n.d.	SRR9924799	16.4	(Liu et al. 2020) Environmental data: (Lu et al. 2023)	7.4	96.6	0.106
Yangtze River, China	30.886945 N 110.905195 156 m	October 2014	7.9	390	n.d.	SRR9924803	17.4	(Liu et al. 2020) Environmental data: (Lu et al. 2023)	1.4	27.7	0.005
Yangtze River, China	29.62 N 106.6 E 155 m	October 2014	8.0	405	n.d.	SRR9924798	14.3	(Liu et al. 2020) Environmental data: (Lu et al. 2023)	11.4	97.7	0.188
Yangtze River, China	31.04 N 110.4 E 63 m	October 2014	7.9	390	n.d.	SRR9924797	19.6	(Liu et al. 2020) Environmental data: (Lu et al. 2023)	2.1	64.2	0.016
Yangtze River, China	30.69 N 111.28 E 36 m	October 2014	7.9	700	n.d.	SRR9924791	12.6	(Liu et al. 2020) Environmental data: (Lu et al. 2023)	1.2	10.4	0.002
Yangtze River, China	30.29 N 112.26 E 28 m	October 2014	7.8	340	n.d.	SRR9924790	15	(Liu et al. 2020) Environmental data: (Lu et al. 2023)	1.3	24.7	0.005
Yangtze River, China	30.62 N 114.32 E 20 m	October 2014	7.8	350	n.d.	SRR9924753	14.1	(Liu et al. 2020) Environmental data: (Lu et al. 2023)	1.7	48.9	0.014

Fig. S1 Figure used to determine if a mapping result on the metagenomes was regarded as positive or negative. It shows mapping of *Polynucleobacter paneuropeus* strains on metagenomes harboring genomes of *Polynucleobacter paneuropaeus* (i.e., mapping results in coverage depth > 100-fold). The number of mapped metagenomic reads was stepwise reduced and the respective coverage depth and breadth were recorded (see main text).

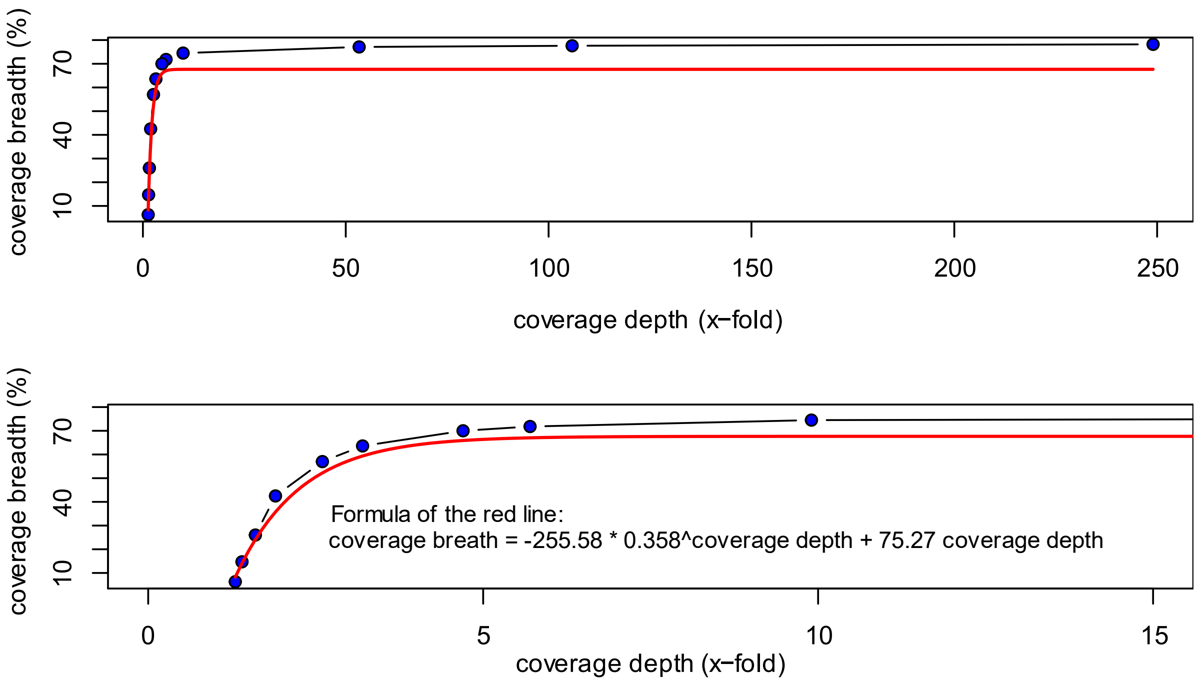

Fig. S2 Polar lipid pattern of strains 1-SAACH-A3^T, 2-BAHN-186B^T, and 2-AUSEE-184A6. For each strain: first row: left side, visualization of total lipids with dodecamolybdophosphoric acid; right side: visualization of glycolipids with α-naphthol Second row: left side, visualization of phospholipids with molybdenum blue; right side, visualization of aminolipids with ninhydrin. F24-239, 1-SAACH-A3^T; F24-241; 2-BAHN-186B^T; F24-240, 2-AUSEE-184A6

PE, phosphatidylethanolamine; APL, aminophospholipid; GL, glycolipid; PL, phospholipid; L, lipid

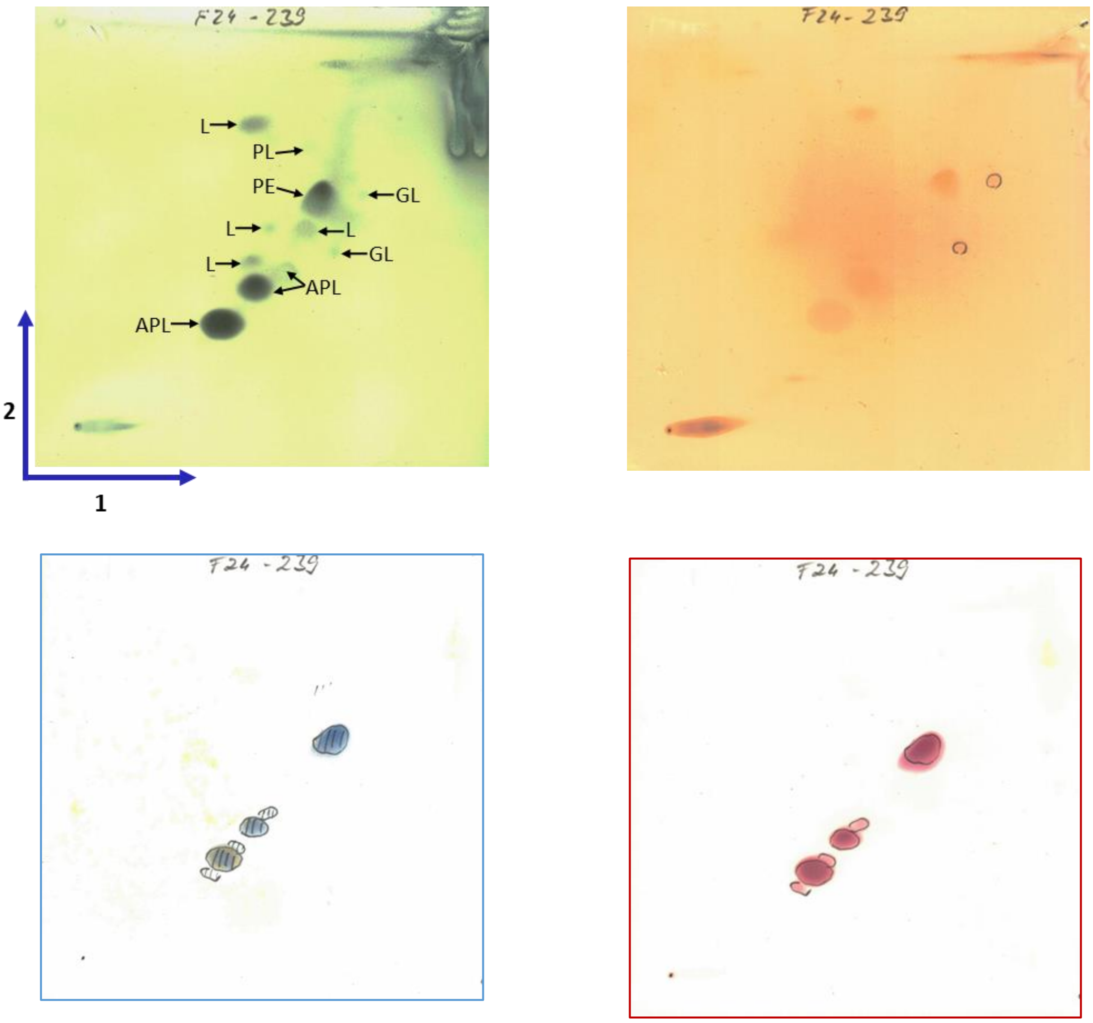

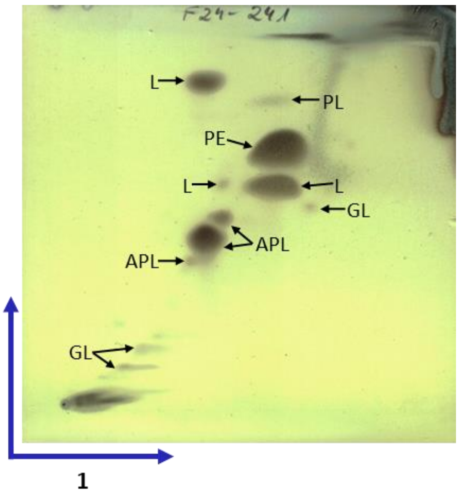

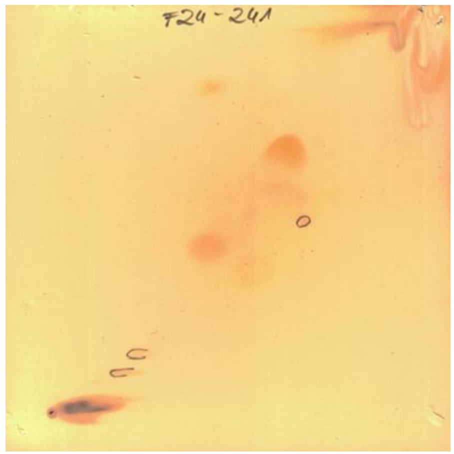

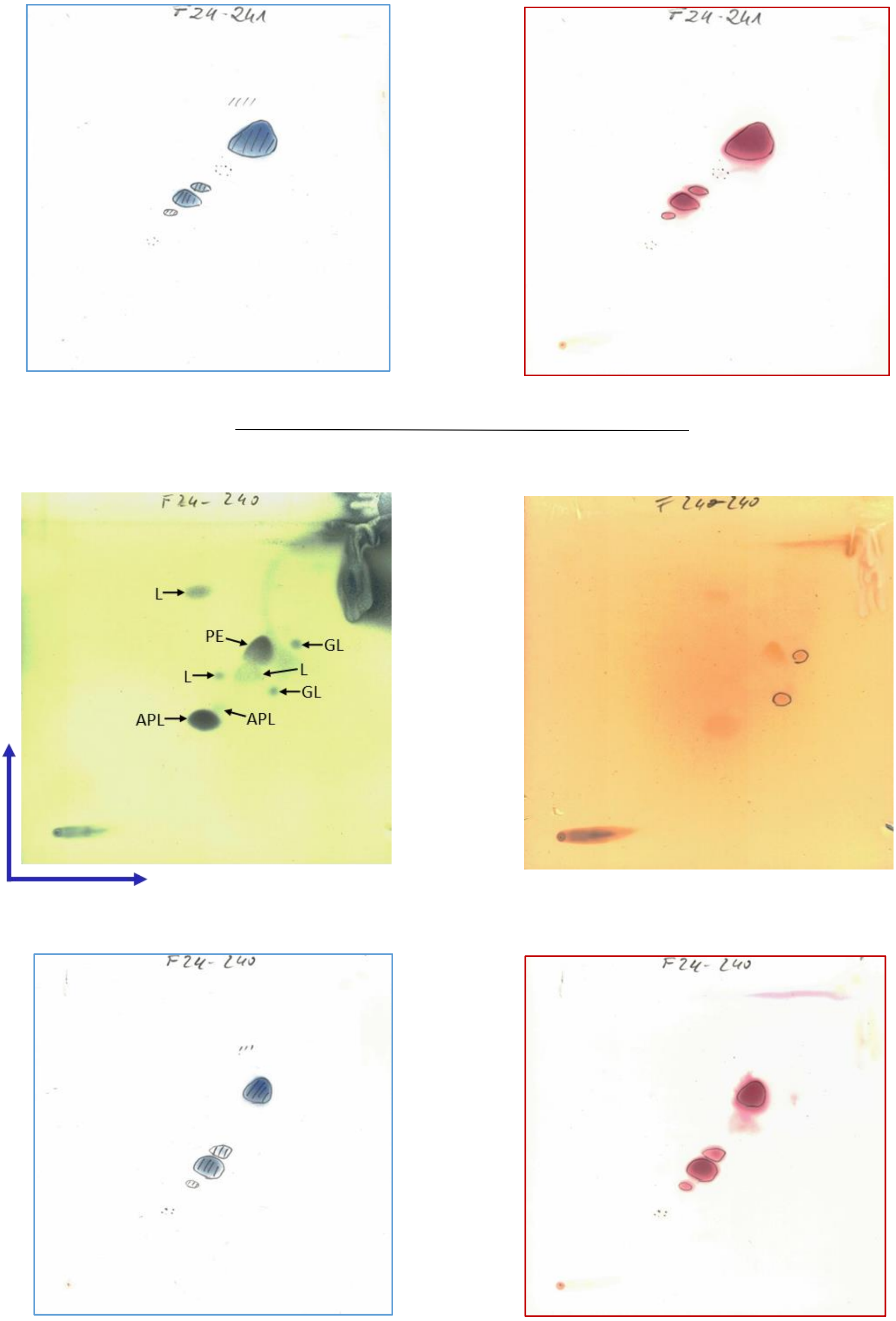
F24-241
F24-241
F24-240
L
PE
GL
L
L
GL
APL
APL
F24-240
F24-240

Fig. S3 Reconstruction of the phylogenetic position of the investigated strains and related strains (same selection as used in Fig. 1) based on almost full-length 16S rRNA gene sequences (1331 alignment positions). Shown is a neighbour-joining midpoint rooted tree.

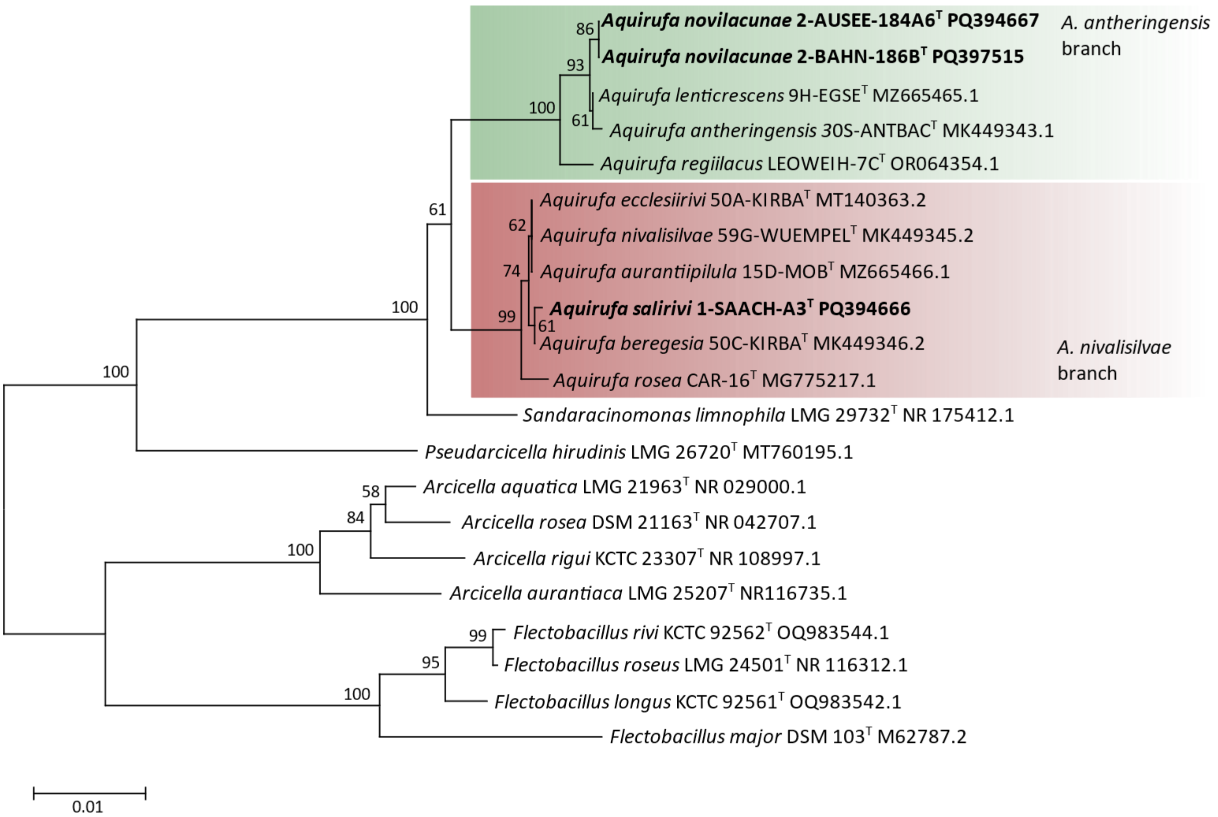

References:

Liu S, Wang H, Chen L et al (2020) Comammox *Nitrospira* within the Yangtze River continuum: community, biogeography, and ecological drivers. The ISME Journal 14:2488-2504. https://doi.org/10.1038/s41396-020-0701-8

Lu J, Gu J, Han J et al (2023) Evaluation of spatiotemporal patterns and water quality conditions using multivariate statistical analysis in the Yangtze River, China. Water 15:3242

Shim MJ, Yoon SC, Yoon YY (2018) The influence of dam construction on water quality in the lower Geum River, Korea. Environ Qual Manage 28:113-121. https://doi.org/10.1002/tqem.21591

Yang Y, Huang X, Zhu X et al (2020) Spatiotemporal characteristics of the water quality in the Jinsha River Basin (Panzhihua, China). Water Supply 21:189-203. https://doi.org/10.2166/ws.2020.258
